# Ultrasound-based Techniques as Alternative Treatments for Chronic Wounds: A Comprehensive Review of Clinical Applications

**DOI:** 10.7759/cureus.1952

**Published:** 2017-12-15

**Authors:** Saad Ahmed Alkahtani, Pramod S Kunwar, Mostafa Jalilifar, Samaneh Rashidi, Ali Yadollahpour

**Affiliations:** 1 Department of Clinical Pharmacy, College of Pharmacy, Najran University, Najran, Kingdom of Saudi Arabia; 2 Department of Pharmaceutics, Modern Institute of Pharmaceutical Sciences, Indore, Madhya Pradesh 453111, India; 3 Department of Medical Physics, Faculty of Medicine, Ahvaz Jundishapur University of Medical Sciences, Ahvaz, Iran; 4 Department of Medical Physics, School of Medicine, Ahvaz Jundishapur University of Medical Sciences, Ahvaz, Iran

**Keywords:** ultrasound, chronic wound, clinical considerations, dose, therapeutic efficacy

## Abstract

Ultrasound (US) waves have been recently developed for the treatment of different chronic wounds with promising therapeutic outcomes. However, the clinical efficacy of these techniques is still not fully understood and standard guidelines on dose ranges and possible side effects should be determined. This paper aims to comprehensively review the recent advances in US techniques for chronic wound treatment, their therapeutic efficacies, and clinical considerations and challenges. The databases of PubMed (1985-2017), EMBASE (1985-2017), Web of Sciences (1985-2017), Cochrane central library (1990-2017), and Google Scholar (1980-2017) were searched using the set terms. The obtained results were screened for the title and abstract by two authors and the relevant papers were reviewed for further details. Preclinical and clinical studies have shown strong evidence on the therapeutic efficiency of US in chronic wounds. The main limitation on developing clinical standard protocols of US for treatment of wounds is the lack of definite dose-response for each wound. However, spatial average temporal average is the main parameter for defining US dosage in wound treatment. The range of 0.5 to 3 W/cm^2^ is a range of dose exerting significant therapeutic outcomes and minimum adverse effects. Low-frequency US waves can accelerate the healing speed of open wounds as well as deep-tissue injuries. In addition, US waves show promising therapeutic efficacy for chronic wounds. To develop clinical US protocol for each wound type, further in vitro and in vivo preclinical and clinical trials are needed to reach an exact dose-response for each wound type.

## Introduction and background

During the recent years, along with the advancements in new medications, different techniques have been developed for the treatment of different chronic wounds such as pressure relieving beds, and medicinal plants [1]. However, high worldwide prevalence of wounds, high costs and side effects of conventional medications have necessitated the development of alternative or adjunctive techniques for wound treatment. In this regard, several methods have been developed for the treatment of different acute and chronic wounds including laser [2], direct current, electric and magnetic fields [3, 4], light and electromagnetic fields [5].

Ultrasound (US) waves have been recently proposed for the treatment of different wounds and showed promising outcomes. These mechanical waves have several intrinsic advantages over other non-medication techniques that make them a good candidate for wound healing. The capability of deep penetration to reach deep-seated tissues, being highly orienting and focusing, and low scattering are some of these advantages [6]. Different US-based techniques have been developed enjoying these features for the treatment of different disorders including skin wounds, musculoskeletal disorders, malignant tumors, and bone fractures [7, 8]. US waves have reportedly shown promising outcomes for soft tissue injuries than other disorders [7]. Several preclinical and animal studies have shown different physiological effects of US on living tissues [9].

In this regards, high-frequency US waves were used in tendon injuries treatment and short-term pain relieving [10], fresh fracture healing [11], venous and pressure ulcers, and surgical incisions [12, 13]. However, some studies have reported possible side effects of US waves under inappropriate parameters that can cause burns or damage the endothelial tissues [1]. In line with the studies on these fields and promising outcomes, different commercial US-based modalities have been developed. Most of these devices work in low frequencies as the use of high-frequency US in clinical medicine is restricted due to the risk of tissue heating. Low-frequency US waves are actually a slow release technique associated with low tissue heating so that these techniques may become the standard technique for delayed healing wounds, skin ulcers, and nonunion fractures. Surface acoustic wave (SAW) patch therapy is another US technique developed for wound treatment. It employs a different acoustic wave than traditional ultrasound, utilizing a scattered beam with a maximum penetration of 4 cm, while traditional ultrasound can penetrate 10 cm. Some studies have reported that the application of SAW patch therapy increases tissue oxygenation and saturation, which consequently facilitates the wound healing [14, 15].

US waves have emerged as a promising alternative or adjunctive strategy for chronic wounds. However, the clinical efficacy of these techniques for different chronic wounds is still not fully understood. In addition, the clinical guidelines on the allowed doses and possible side effects of these techniques should be determined. To address this issue, the present study was aimed to review the therapeutic effectiveness of US-based techniques for treatment of chronic wounds and the clinical challenges for the development of these techniques as routine approaches for wound treatment are discussed.

## Review

Method

The databases of PubMed (1985-2017), EMBASE (1985-2017), Web of Sciences (1985-2017), and Google Scholar (1980-2017) were searched using the set terms. The title and abstract of the obtained records were reviewed by two authors and they came to the consensus whether the studies are related to the review. Animal and human studies in both in vivo and in vitro designs that evaluate the therapeutic effects of US waves in chronic wounds were included for further review. Because of the immense body of literature in this field, various protocols and devices used in different wound types, this study was aimed to provide a comprehensive and descriptive overview of the recent advances in applications of US waves for the treatment of chronic wounds, therapeutic efficacies, and clinical considerations of US-based techniques for chronic wounds.

Search strategy

Scientific records were retrieved using a systematic searching of multiple bibliographic databases. The last update of the search was performed on May 30th, 2017 including PubMed (1985-2017), EMBASE (1985-2017), Web of Sciences (1985-2017), and Google Scholar (1980-2017). The language of search was limited to English. The search key words based on the MeSH heading included "ultrasound wave" OR "ultrasound" AND "chronic wound" AND "treatment" OR "clinical considerations" OR "therapeutic efficacy" OR "dose response". The titles and abstracts of all the records retrieved by the search strategy were carefully reviewed by at least two authors and the relevant records with full texts available were used for further assessments. In addition to the records identified in the systematic search of databases, the reference lists of the relevant papers were assessed manually to identify studies appropriate for the full reviews and the eligible studies were also included in the full review stage.

Inclusion and exclusion criteria

**Figure 1:**
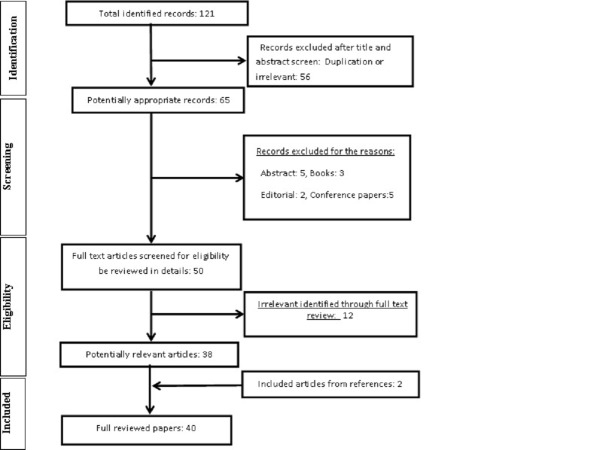
Flowchart of the study procedure.

The identifying, screening, and eligibility stages of studies for inclusion or exclusion were performed independently by the three authors and disagreements were resolved by discussion. Those studies that presented an original research and the main criterion for eligibility of a study were human or animal studies in vivo or in vitro assessing therapeutic outcomes or a biological effect of US waves in any types on any kind of chronic wounds. The exclusion criteria were: (1) abstract only, (2) books, (3) letters, (4) conference papers, (5) editorials or (6) guidelines assessments (Figure 1).

Results

A total of 121 studies were retrieved from the searching process. After screening the abstracts and titles, 65 records remained. In the screening stage, 15 records were removed as being abstract only (5), book (3), editorial (2), and conference records (5). In the eligibility stage, 12 records were removed and 38 papers were included for full-text review. Two more papers were also added from the reference lists screening, and totally, 40 papers were included in the final review.

Physical characteristics of therapeutic US

The US waves delivered to the body and soft tissues undergo diffusion and through vibrating, the molecules progressively lose their energy as the waves pass through the tissue. The main phenomena causing the US waves attenuation are absorption, scattering or dispersion, reflection, and rarefaction [16]. Power expressed in Watts is the main parameter to assess the therapeutic outcomes of US-based techniques. The amount of energy transferred to a target tissue is determined by two main groups of parameters: the US waves' characteristics (frequency, intensity, amplitude, focus, and beam uniformity) and the type and physical characteristics of the target tissue as well as tissues the US waves pass them. The therapeutic US waves have a frequency range of 0.75–3 MHz and most of the US devices are set at two frequencies: 1 or 3 MHz. Increasing the frequency decreases the penetration depth; however, low-frequency US waves are less focused. One-MHz US waves are adsorbed mainly by tissues located in depth of 3–5 cm making them an appropriate option for deeper injuries and in patients with higher content of subcutaneous fat. In contrast, 3 MHz US waves are appropriate for more superficial lesions at depths of l–2 cm [17]. Regarding medical applications, acoustic impedance is the main parameter describing a tissue, which is defined as the product of the tissue's density and the US wave's speed within the tissue. Tissues that are rich in fat have low US absorption and thus high penetration of US waves, whereas tissues with high protein content such as skeletal muscle have higher US adsorption and low penetration. The acoustic impedance difference between two tissues determines the percent of reflection at the interface of the two tissues where higher differences mean lower percents of transmission through the interface [16]. When reflected US meets further transmitted waves, a standing wave is generated that may impose adverse effects on tissue. These adverse effects can be minimized through producing a uniform wave, using pulsed waves, and moving the transducer during the treatment intervention.

Transducers with greater diameter produce more focused US beam. Energy distribution within the US beam is not uniform and the greatest non-uniformity is formed near the transducer surface. The variation of the beam intensity is determined by the beam non-uniformity ratio (BNR), defined as the ratio of the maximum intensity of the transducer to the averaged intensity across its surface.

Mechanisms of action

In vitro studies have shown the therapeutic outcomes of US waves on tunneling or debilitation wounds are mainly through killing multi-drug resistant bacteria such as vancomycin-resistant Enterococcus and resistant Pseudomonas aeruginosa [18]. Several in vitro studies have shown that US waves improve cell proliferation, collagen production, bone formation, and angiogenesis [17]. One of the proposed mechanisms of action of US waves in wound treatment is reducing the wound-related pains. Pain associated with chronic wounds is always a challenging clinical issue with no definitive solution. Different studies have demonstrated the therapeutic efficacy of low-frequency US waves in chronic wounds, not only for curing but also for pain relieving, decreasing pigmentation and odor [19]. Clinical evidence has shown that the US intervention reduces wound-associated pain in the patients with painful chronic lower-extremity wounds [20]. However, there are controversial findings on the clinical outcomes of US waves. For instance, a systematic review of the efficacy of different US modalities on chronic wound treatment concluded inadequate evidence for clinical efficacy of therapeutic US in chronic wounds [1].

Clinical considerations

Pressure Ulcers

Several studies have been conducted to investigate the efficacy of different US waves in the treatment of pressure ulcers. In general, the evidence for the effectiveness of US waves for pressure ulcers is limited. Some randomized controlled trials conducted on US treatment in pressure ulcers showed no significant differences between the treatment groups [21, 22]. Similarly, Reddy, et al. in a randomized controlled clinical trial found no significant therapeutic efficacy for the US waves in the treatment of pressure ulcers. Flemming, et al. in a systematic review found no vigorous evidence on the therapeutic efficacy of US in the pressure ulcers. However, the main limitations of their study were inconsistency in the research methodology and physical parameters of the reviewed studies and the small sample size of the reviewed studies [23]. Akbari Sari, et al. reviewed the effectiveness of US therapy on pressure ulcers. They showed no reliable proof of advantage of treatment by US in the healing of pressure ulcers. However, their review suffered the heterogeneities in the research methods and the small sample size of the reviewed studies [23]. Therefore, to reach a definitive conclusion on the therapeutic efficacy and clinical value of US waves in the treatment of pressure ulcers further studies should be conducted.

Combined US - Traditional Techniques

Low-frequency US techniques have been used in combination with standard wound care medications for treatment of purulent wounds. The findings of these studies showed the therapeutic effectiveness of US technique as an adjunctive or alternative treatment for purulent wound. A case series study (n = 17) showed the effectiveness of the combination of low-frequency US together with gentamicin solution so that the purulent septic complications were reduced from 35.7% to 5.9% [24]. Several studies have investigated the efficacy of low-frequency US in combination with antibiotic agents in different chronic wounds or bacterial cultures. Rediske, et al. demonstrated that continuous US waves and systemic gentamicin administration significantly decreased the viable bacteria concentration in the simulated implant putridity [25]. Other studies have reported that application of US in the bacterial cultures of E. coli and P. aeruginosa increased the efficacy of antibacterial action of gentamicin [26, 27].

Diabetic Wounds

A cross-sectional experiment comparing the therapeutic outcomes of low-frequency US and laser irradiation in patients with diabetes mellitus and purulent surgical wound (n = 112) reported higher-effectiveness of the US treatment in the first and second phases of wound healing process [28]. Swist-Chmielewska, et al. compared the efficacy of US waves at two power densities of 0.5W/cm^2^ and 1 W/cm^2^ for the treatment of Venous crural ulceration [29]. They reported US waves speed up the ulceration healing process and US waves at 0.5 W/cm^2^ showed greater outcomes. However, they reported no significant difference in terms of granulation development rate and debridement of the wound between the two densities.

Gottrup and Apelqvist carried out a review of the available literature on new methods for the treatment of diabetic foot ulcers [30]. They evaluated the therapeutic efficacies of several wound healing techniques including antimicrobial agents, dressings, topical negative pressure, hyperbaric oxygen treatment, electrical, electromagnetic, laser, shockwave, and US techniques, growth, and cell biology modulating factors, tissue engineering, bioengineered skin and skin grafts, and adjuvant therapies. Their review demonstrated a restricted proof on the level I evidence to recommend these techniques as usual clinical methods. However, some of the US-based techniques can be used as alternative or adjunctive treatment for some types of chronic wounds. The main reasons for the lack of strong evidence are insufficient sample size, short follow-up period, non-random allocation to treatment arms, non-blinded outcomes evaluation, poor description of control, and concurrent interventions. Therefore, it is necessary to enhance the quality and methodology of clinical trials [30].

Extremity Lower Wounds

Extremity lower wounds are the most prevalent wounds worldwide and most of the US-based techniques have been proposed for this type of wounds. Johannsen, et al. in a meta-analysis on the efficacy of US waves in the treatment of chronic leg ulcer concluded that low doses of US administered around the ulcer edge exert the greatest therapeutic effects [31]. Callam, et al. compared the therapeutic outcomes of standard wound care with a pulsed US intervention for a 12-week period for chronic leg wounds. They reported that the ratio of wound closure area was 20% greater in the US intervention group [32]. Lundeberg, et al. in a randomized controlled trial (n = 44) investigated the outcomes of a combined treatment of pulsed US and a standard wound healing technique for chronic leg ulcers [33]. They compared the outcomes of a standard treatment (paste impregnated bandage and a self-adhesive elastic bandage) with a placebo US and with real pulsed US intervention applied three times a week for four consecutive weeks, followed by two times a week for other four-week period and then once a week for next four weeks. The wound healing rates were assessed after four, eight and 12 weeks [33]. They observed no significant differences in the percentage of cured ulcers in the pulsed US treatment as compared with the placebo group [33].

Eriksson, et al. in a randomized controlled trial compared the therapeutic efficacy of a US treatment consisting of 1.0 W/cm^2^ at 1 MHz, for 10 min twice a week for eight weeks in a standard model of chronic leg ulcers. They observed no significant differences between the real and placebo treatments in the percentage wound closure area and the number of cured wounds examined at two, four, six, and eight weeks after the start of treatment [34]. Peschen, et al. investigated the outcomes of low-frequency (30 kHz) low-intensity US on the chronic venous leg ulcers combined with a conventional outpatients’ therapy. Patients were randomly divided into two groups: conventional treatment with topical application of hydrocolloid dressings and combined US-conventional treatment (compression therapy). The US therapy consisted of a 10-min of foot-bathing with continuous US wave at 100 mW/cm^2^ density three times a week for three months. The ulcer area was measured before intervention and after intervention at two, four, six, eight, 10, and 12 weeks post-intervention. In addition, the radius of ulcer was measured daily. After each session, adverse effects were evaluated. The results showed the mean decrease of ulcer area in the US group was 55.4% compared to 16.5% for the control group. In addition, daily decrease of ulcer size in the US-treated group was 0.08 mm compared to 0.03 mm for the control. Both US and control groups indicated minor adverse effects. The findings of this study confirmed the therapeutic efficacy of the low-frequency low-dose US technique in chronic venous leg ulcers [35].

The American Society of Plastic Surgeons evaluated the efficacy and feasibility of US treatment for leg and foot ulceration [36]. Their assessments, which were based on the clinical experiment guidelines on chronic wounds of the leg and foot ulcer, did not note the application of US as a choice of treatment [36]. Kavros, et al. in a retrospective analysis evaluated the clinical efficacy of MIST-US technique for chronic leg and foot ulcer. They reported that the efficacy of wound healing of a standard wound care in combination with MIST-US technique was significantly higher than the standard wound care alone. In addition, application of the MIST-US therapy accelerated the wound healing rate compared with the standard wound care [14].

Cullum, et al. conducted a Cochrane review on the efficacy of US on the rate of venous leg ulcer healing. They concluded that the conducted studies on US treatment in venous leg ulcers suffer small clinical evidence with low sample size, poor-quality, and heterogeneous. Their review concluded no significant evidence of the efficacy of US for venous leg ulcers healing. There was a number of weak evidence which showed enhanced therapeutic efficacy of US; however, to reach a more conclusive answer, further high-quality large sample size randomized controlled trials are needed [20].

Low-Frequency Non-Contact US

One of the new US-based techniques for the wound treatment with promising outcomes is non-contact low-frequency US (NLFU) techniques which is also called MIST-US treatment. This technique has been approved by the United States Food and Drug Administration for wound treatment [37]. Several case-series, preclinical, and randomized controlled trials have been conducted on the efficacy of NLFU in the treatment of different chronic wounds including burns, digital ulcers, infected surgical wounds, and sacral pressure ulcers [38-44]. In a randomized, double-blinded, sham-controlled, multi-center study, Ennis, et al. compared the therapeutic efficacy of active and sham NLFU (40 kHz) US technique using a MIST-US device in recalcitrant diabetic foot wounds therapy (n = 55). The active US treatment showed a significant increased wounds healing ratio compared to the placebo group. Furthermore, the frequency and type of the reported side effects did not differ between the two groups [45]. Kavros, et al. investigated the effects of NLFU therapy on ischemic wounds and reported significant improvements in wound healing period in patients with critical limb ischemia following the administration of combined standard wound care and NLFU [46]. The treatment protocol consisted of daily five-minute treatment for three times a week for consecutive three months or until wounds reached a full recovery. The main outcome of remission was defined a more than 50% wound area reduction after three-month treatment period. The percentage of cured patients in the combined standard wound care and MIST-US group (63%) was significantly higher than the standard wound care group (29%). This study also proposed that the baseline transcutaneous oxygen pressure is a parameter that can predict the outcome of US waves on wound healing [46].

Kavros and Schenck investigated the efficacy of NLFU treatment in chronic, intractable lower-leg and foot ulcer (n = 51) using a non-randomized, baseline-controlled clinical series [15]. They compared the efficacies of a standard wound treatment and low-frequency US therapy alone and in combination. The patients had leg and foot ulcer different etiologies including diabetes mellitus, neuropathy, limb ischemia, chronic renal insufficiency, venous illness, and inflammatory connective tissue disease. The average wound healing period for the baseline standard wound care, control group, was 9.8 weeks, whereas NLFU group showed healing period of 5.5 ± 2.8 weeks. They concluded NLFU treatment can significantly improve the wound healing in recalcitrant leg and foot ulcer [15]. Ennis, et al. investigated the efficacy of MIST-US on the wound closure in chronic non-healing lower extremity wounds with different etiologies. They showed appropriate and optimal treatment duration can enhance the wound healing process and the improvements were clinically significant [47].They also investigated the effect of MIST-US on the micro circulatory flow patterns within the wound bed. The standard treatment period was two weeks and 69% of the wound was healed by applying the desired therapeutic model. When MIST US was applied alone, the average wound healing period was reduced to seven weeks, compared with the 10-week healing period in the control group. They concluded that using MIST-US treatment alone or in combination with moist wound care could completely heal 69% of chronic wounds [47].

## Conclusions

The preclinical in vitro and in vivo studies along with clinical studies show that US waves in specific frequencies, mainly low-frequency range, can shorten the healing period of open wound. In addition, these waves can be clinically effective for early treatment of deep-tissue injuries. Although early studies have been relatively promising, the main challenge for developing US-based techniques as standard treatment options for different wound is defining an exact dose-response for each wound. One of the main steps to define the dose response for US applications in wound treatment is defining the exact mechanisms of action as a function of main physical parameters of US waves as well as biological parameters of the target wounds. In this regard, conducting further controlled trials with big sample size is necessary to reach this goal.
